# Mechanistic diversity of clamp loading at small DNA gaps

**DOI:** 10.1016/j.jbc.2026.113397

**Published:** 2026-08-10

**Authors:** Fengwei Zheng, Michael E. O’Donnell, Huilin Li

**Affiliations:** 1 Department of Structural Biology, Van Andel Institute, Grand Rapids, Michigan, USA; 2 DNA Replication Laboratory and Howard Hughes Medical Institute, The Rockefeller University, New York, New York, USA

**Keywords:** 9-1-1 clamp, clamp loaders, cryo-electron microscopy, Ctf18-RFC, DNA bending, DNA gaps, DNA repair, DNA replication, DnaX complex, Elg1-RFC, PCNA, β-clamp, Rad24-RFC, replication factor C, sliding clamps

## Abstract

DNA sliding clamps, including PCNA (proliferating cell nuclear antigen) and the 9-1-1 (RAD9–RAD1–HUS1 in humans) complex, are ring-shaped protein complexes that encircle DNA and serve as central interaction platforms in DNA replication, repair, and checkpoint signaling. While clamp loading at canonical primer–template junctions by AAA+ (ATPases associated with diverse cellular activities) clamp loaders is well established, how clamps are loaded onto physiologically relevant but geometrically constrained DNA intermediates, such as nicks and single-stranded gaps, has remained unclear. Recent cryo-electron microscopy studies reveal that clamp loaders have evolved distinct strategies to overcome these constraints and to specialize for different genomic contexts. At gapped DNA, the eukaryotic clamp loader RFC (replication factor C) engages both 3′- and 5′-recessed DNA ends and can locally unwind DNA, enabling PCNA loading across a wide range of gap sizes. In contrast, the bacterial DnaX clamp loader lacks a 5′-DNA-binding site and does not unwind DNA, instead loading the β-clamp at small gaps (<6 nt) by sharply bending DNA. The checkpoint clamp loader Rad24-RFC (RAD17-RFC in humans) similarly lacks DNA unwinding activity, restricting loading of 9-1-1 clamp to larger gaps (≥6 nt). In a distinct specialization, Ctf18-RFC interacts with the leading-strand DNA polymerase ε, positioning it as a dedicated loader for leading-strand synthesis, whereas Elg1-RFC (ATAD5-RFC in humans) excludes DNA from its chamber and functions as a PCNA unloader. Together, these mechanisms illustrate how clamp loaders are diversified to accommodate DNA structure and replisome context, ensuring coordinated control of genome replication and maintenance.

DNA sliding clamps are evolutionarily conserved ring-shaped protein complexes that encircle DNA and slide along duplex DNA once loaded by clamp loaders. They function as central interaction platforms in DNA replication, damage repair, and other DNA metabolic processes (1, 2, 3, 4). Eukaryotes encode two types of clamps: the sliding clamp PCNA (Proliferating Cell Nuclear Antigen), a homotrimer in which each subunit contains two domains, and the DNA damage checkpoint clamp 9-1-1, a heterotrimer composed of RAD9–RAD1–HUS1 in humans (or Ddc1–Rad17–Mec3 in yeast), with each subunit adopting a similar fold. In contrast, prokaryotes such as *Escherichia coli* encode a single sliding clamp, the β-clamp, which forms a homodimer with three domains per subunit (2). Despite these differences, all clamps assemble into pseudo–six-fold symmetric, topologically closed rings that encircle DNA (5, 6, 7, 8, 9) (Fig. 1).

**Figure 1:**
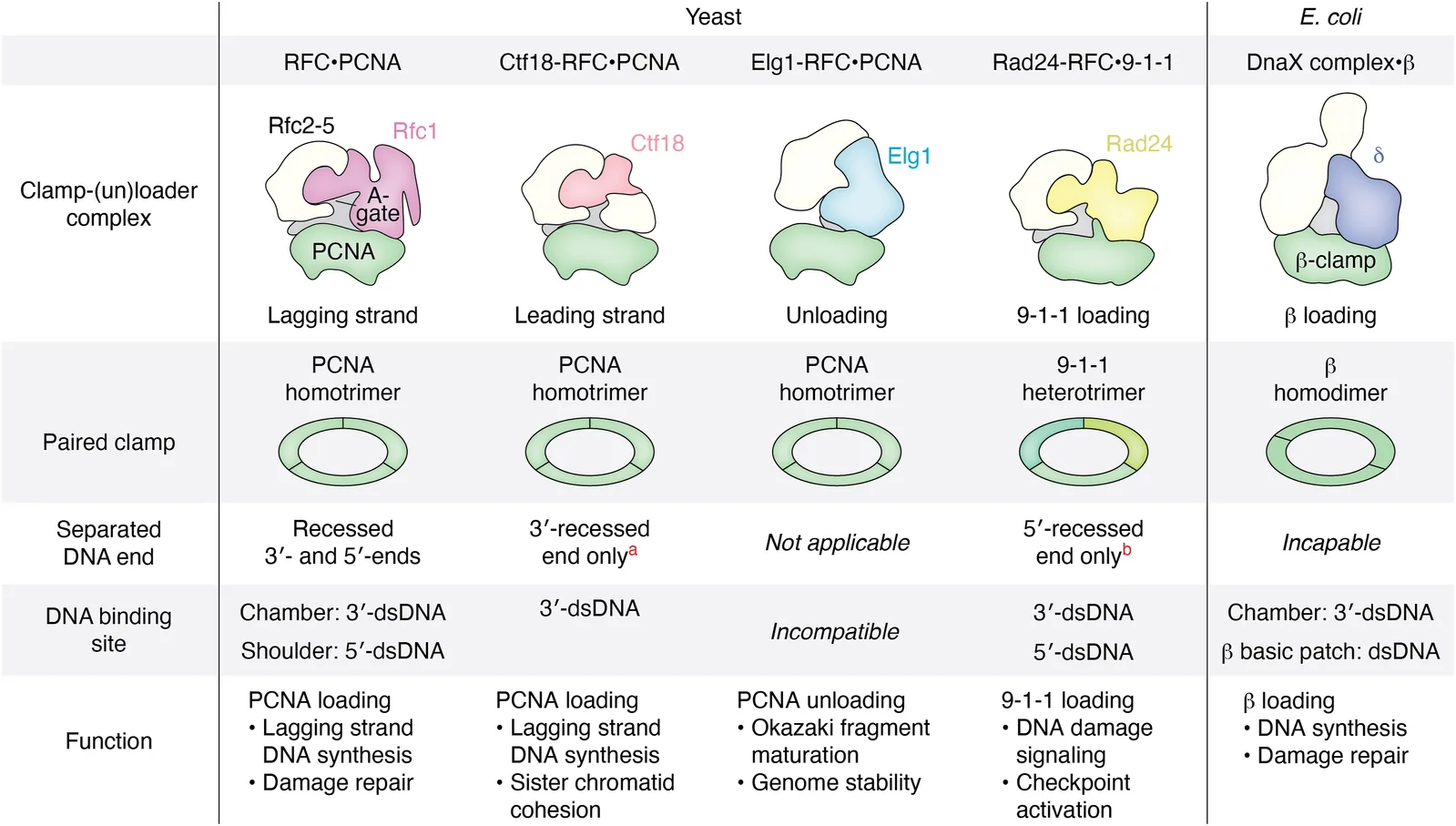
**Comparison of prokaryotic and eukaryotic clamp (un)loading systems.** Schematic overview of the clamp loader complexes discussed in this review, including yeast RFC–PCNA, Ctf18-RFC–PCNA, Elg1-RFC–PCNA, and Rad24-RFC–9-1-1, together with the *E. coli* DnaX complex–β clamp. Rows summarize overall architecture, clamp identity and oligomeric state, DNA separation activity, DNA binding site organization, and representative biological functions. Elg1-RFC is incompatible with DNA binding, whereas the *E. coli* DnaX complex lacks a shoulder DNA-binding site and instead uses a basic patch on the β-clamp for dsDNA interaction. The superscript “a” indicates that the Ctf18 separation pin is inactive in yeast but active in humans. The superscript “b” indicates that Rad24-RFC contains an external separation pin capable of unwinding one base pair at the 5′-recessed DNA end (Castaneda *et al.*, 2022).

Sliding clamps are essential for processive DNA synthesis. The β-clamp and PCNA are loaded at the 3′ end of primer–template (p/T) junctions to recruit replicative DNA polymerases and tether them to DNA, thereby greatly enhancing the rate and processivity of DNA synthesis (10, 11, 12, 13, 14, 15, 16, 17, 18). In contrast, the 9-1-1 clamp is loaded at recessed 5′ ends to recruit the ATR kinase and activate checkpoint signaling pathways that coordinate DNA repair (19). In addition, clamps can be post-translationally modified to regulate their interactions with downstream factors; for example, ubiquitinated PCNA recruits translesion synthesis polymerases, whereas SUMOylated PCNA, in which a small ubiquitin-like modifier (SUMO) is covalently attached to PCNA, suppresses recombination during normal DNA replication (20, 21, 22).

Structural studies have shown that sliding clamps form stable, closed ring structures with central channels large enough to accommodate duplex DNA (5, 6, 7). Although some local flexibility is observed, these rings are highly stable in solution and do not spontaneously open, necessitating the action of ATP-dependent clamp loaders to open a ring and load it onto DNA (23, 24, 25, 26). Clamp loaders are pentameric AAA+ (ATPases associated with diverse cellular activities) complexes composed of ATPase domains, helical lid domains, and a C-terminal collar that forms a closed ring (27, 28). Crystallographic studies established a conserved architecture in which a spiral AAA+ module engages DNA, while a lateral A-gate in the large subunit allows DNA entry into the central chamber (29, 30, 31, 32, 33, 34, 35, 36, 37, 38) (Fig. 1).

Eukaryotes have evolved multiple clamp loaders to fulfill distinct cellular functions. In addition to the canonical Replication factor C (RFC; Rfc1-5), three alternative RFC-like complexes—Ctf18-RFC, Rad24-RFC, and Elg1-RFC in yeast and CTF18-RFC, RAD17-RFC and ATAD5-RFC in humans—share four smaller subunits (Rfc2-5) but differ in their large subunit, which confers functional specialization (28, 39, 40). RFC primarily loads PCNA during lagging-strand DNA synthesis (41), whereas Ctf18-RFC has been implicated in leading-strand synthesis, sister chromatid cohesion, and replication checkpoint signaling (42, 43). Rad24-RFC functions as a checkpoint clamp loader that specifically loads the 9-1-1 complex onto recessed 5′ DNA ends (44, 45, 46, 47, 48), while Elg1-RFC acts as a PCNA unloader to remove clamps from DNA following replication or repair (49, 50). In bacteria, a single clamp loader, the DnaX complex, carries out clamp loading and coordinates replication fork assembly through interactions with DNA polymerase III and single-stranded DNA binding protein (29, 31, 51, 52, 53, 54, 55, 56, 57) (Fig. 1).

Earlier crystallographic studies established a general mechanism in which the clamp loaders open sliding clamps and position the primer 3′ end within their central chambers, allowing the clamp to close around duplex DNA at a p/T junction (5, 29, 31, 32). However, many physiologically relevant DNA intermediates, such as nicks and single-stranded gaps, present additional geometric challenges. These substrates contain both 3′- and 5′-recessed ends and two duplex regions (a 3′-dsDNA and a 5′-dsDNA), while the clamp loader architecture restricts entry of 5′-dsDNA through a narrow tunnel that accommodates only a limited length (∼6 nt) of single-stranded DNA. How clamp loaders recognize and process such substrates, and whether they can efficiently load clamps onto very small gaps, remain unclear.

Advances in cryo-electron microscopy (cryo-EM) have enabled high-resolution visualization of clamp loading on these complex DNA substrates. These studies reveal that different clamp loaders have evolved distinct and sometimes unexpected mechanisms to engage gapped or nicked DNA. In this review, we summarize recent structural and biochemical insights into clamp loading and unloading in both eukaryotic and bacterial systems, with an emphasis on how diverse clamp loaders adapt to DNA geometry and cellular context to perform their specialized functions (33, 34, 35, 36, 37, 38, 43, 51, 52, 58, 59, 60, 61, 62, 63, 64, 65, 66, 67).

## Gap-size-independent PCNA loading by RFC

**Figure 2:**
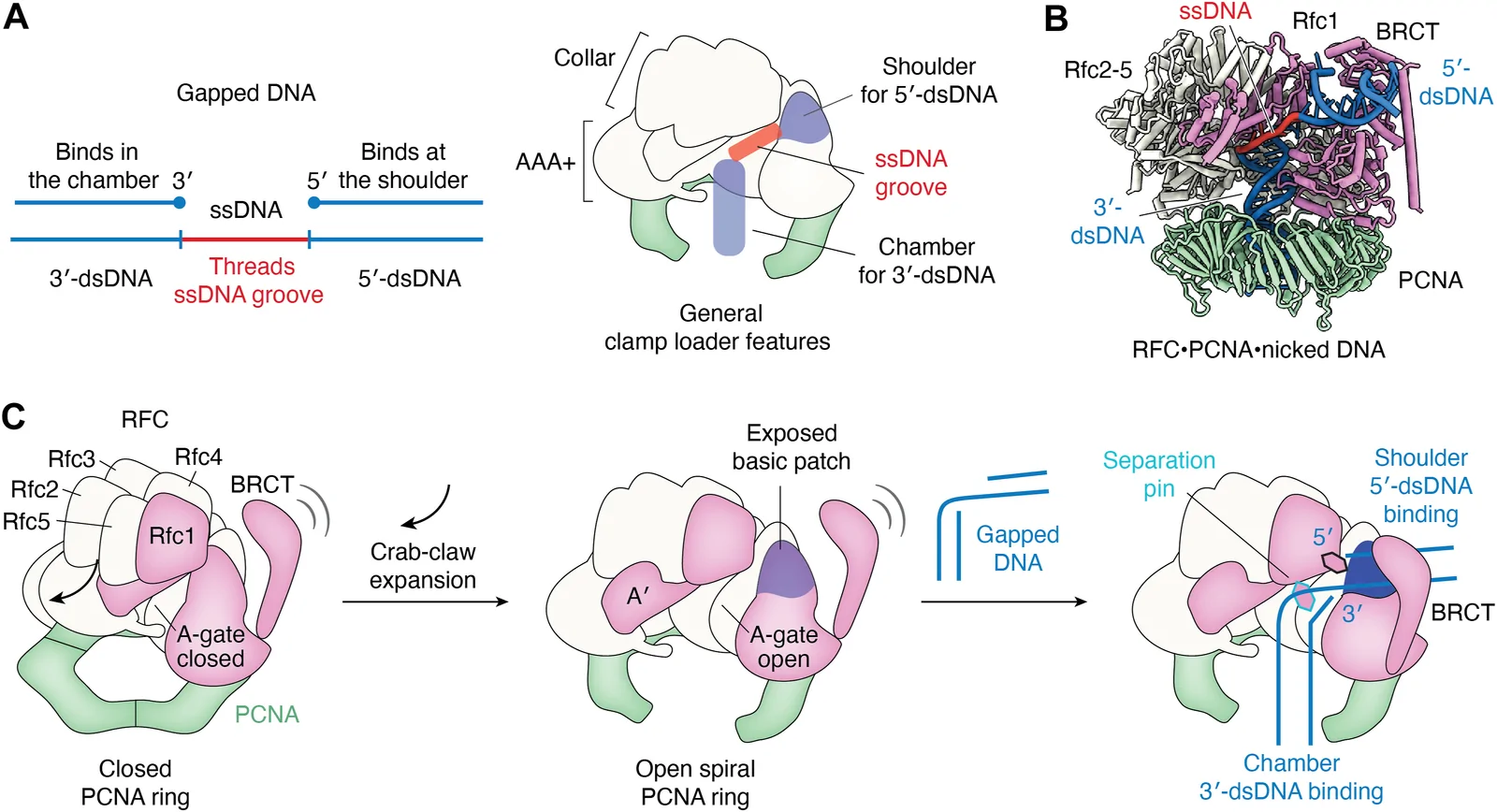
**Simplified model for RFC loading PCNA onto DNA with different gap sizes.***A*, schematic of gapped DNA substrates (*left*) and the RFC–PCNA complex highlighting key structural features (*right*). The 3′-dsDNA segment (dsDNA with 3′-recessed end) binds in the clamp loader chamber, whereas the 5′-dsDNA segment (dsDNA with 5′-recessed end) engages the shoulder site of Rfc1. The intervening ssDNA passes through a narrow groove within RFC enabling simultaneous engagement of both DNA-binding sites. For DNA gaps ≥ 6 nt, the ssDNA can thread the groove directly and span both binding sites, whereas for gaps < 6 nt, local DNA melting is required to support clamp loading. *B*, structure of RFC–PCNA complexed with nicked DNA, in which, three bp are unwound at the 3′-end and at least 1 to 2 bp melting is observed at the 5′-recessed end (PDB 7U19), to convert a nick to a 4-5-nt gap that enables 3′-dsDNA to bind in the chamber and 5′-dsDNA to bind the shoulder, simultaneously. Note that the Rfc1 BRCT domain sandwiches the 5′-dsDNA over the shoulder binding site for further stabilization. *C*, cartoon illustrating conformational changes during RFC-mediated PCNA loading. ATP-bound RFC initially associates with closed PCNA in an autoinhibited state. Subsequent clamp opening through a crab-claw expansion exposes the Rfc1 shoulder DNA-binding site and enables coordinated binding of 3′- and 5′-dsDNA segments within the chamber and shoulder sites, respectively. RFC separation pin residues promote local DNA unwinding to widen small gaps and facilitate simultaneous DNA binding.

The crystal structure of the yeast RFC–PCNA complex revealed that ATPγS binding induces a spiral arrangement of the AAA+ modules that matches the helical pitch of DNA, although DNA-bound structures had not yet been reported at the time (30). Recent cryo-EM studies have now captured RFC–PCNA complexes with p/T DNA and gapped DNA, revealing multiple intermediates along the clamp loading pathway (33, 34, 35, 36). Together with previous crystal structural studies (31, 32), these studies revealed three striking features of clamp loaders, *that is*, a primary 3′-dsDNA binding site located within the internal chamber of the clamp loader, a secondary 5′-dsDNA binding site located on the external shoulder of the clamp loader, and a narrow groove that accommodates only ssDNA, thereby connecting the 3′- and 5′-dsDNA segments (Fig. 2, *A* and *B*).

In these structures, RFC bound to closed PCNA adopts an autoinhibited state, in which only Rfc1, Rfc3, and Rfc4 engage the clamp (38) (Fig. 2*C* Left). Clamp loading proceeds through coordinated conformational changes driven by ATP binding. RFC first engages PCNA and undergoes a “crab-claw” expansion that opens both the clamp loader and PCNA, accompanied by rotation of the AAA+ modules and opening of the Rfc1 A-gate. Partial unfolding of the Rfc1 AAA+ lid creates a transient pore between the AAA+ module and collar domain, allowing DNA entry into the central chamber. In this open state, all five RFC subunits contact PCNA, stabilizing a clamp with an expanded gate (∼20 Å) (Fig. 2*C* Middle).

DNA binding occurs in a stepwise manner. The 3′-recessed end of the p/T DNA is first captured within the RFC chamber, where the Rfc5 β-hairpin (E-plug) inserts into the major groove and stabilizes both DNA strands. An aromatic residue in Rfc1 (W638) functions as a separation pin to locally unwind the duplex and position the primer terminus. As DNA engagement proceeds, PCNA closes around the duplex, while ATP hydrolysis is triggered at a late stage to promote RFC dissociation, leaving PCNA topologically linked to DNA.

A key advance from recent studies is the discovery of a second DNA-binding site for 5′-dsDNA (34, 35, 36) (Fig. 2*C* Middle and Right). Upon clamp opening, a basic patch on the Rfc1 AAA+ lid, together with the N-terminal BRCT domain, forms an external “shoulder” site that captures the 5′-dsDNA, extending earlier observations from Rad24-RFC (59, 62). This dual-site architecture enables simultaneous engagement of both 3′- and 5′-recessed ends of gapped DNA. Additional aromatic residues, including H659, may facilitate limited strand separation at the 5′ end to accommodate short gaps (36).

These findings support a unified mechanism in which RFC loads PCNA onto gapped DNA by first capturing the 3′-end, then engaging the 5′-end *via* the shoulder site, and, when necessary, locally unwinding DNA to widen the gap. This ability to coordinate dual DNA binding and strand separation allows RFC to load PCNA across a wide range of gap sizes, thereby supporting both DNA replication and repair.

## Specialized PCNA loading by Ctf18-RFC for Pol ε–mediated leading-strand synthesis

**Figure 3:**
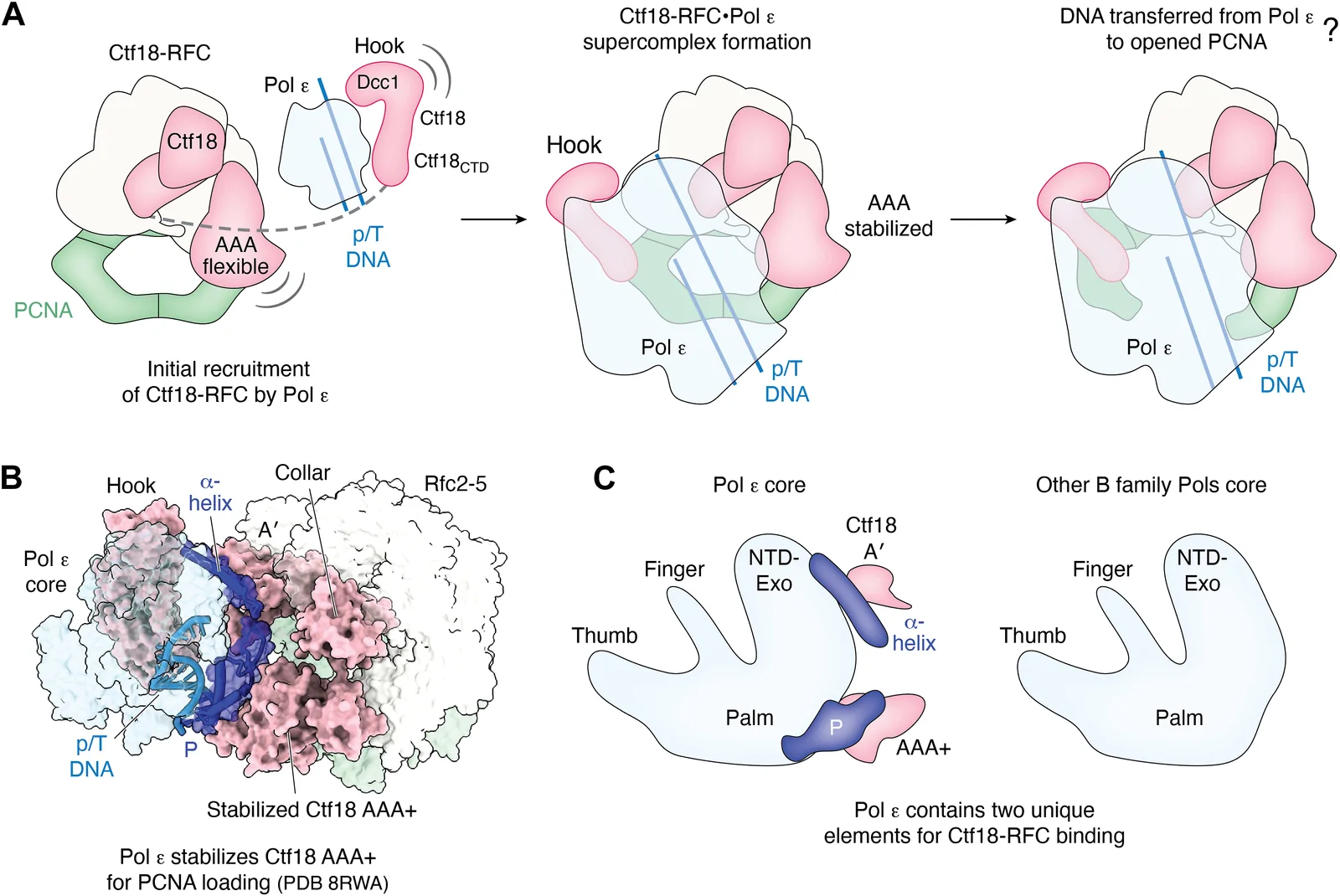
**Ctf18-RFC couples PCNA loading to the leading strand polymerase.***A*, model illustrating key steps of Ctf18-RFC-mediated PCNA loading at the leading strand. First, the Ctf18_CTD_–Ctf8–Dcc1 hook module tethers Ctf18-RFC to Pol ε. Second, interactions between Ctf18-RFC and Pol ε–PCNA–DNA complex align the DNA substrate with PCNA gate. Third, DNA is laterally transferred from Pol ε into the opened PCNA ring. *B*, cryo-EM map of an experimentally captured intermediate, highlighting interactions between the Pol ε P domain and N-terminal α-helix with the AAA+ and A′ domains of Ctf18 that stimulate Ctf18-RFC clamp-loading activity. *C*, structural comparison of Pol ε with other B-family polymerases, highlighting unique features that mediate interaction with Ctf18-RFC. Together, these schematics illustrate how Ctf18-RFC is physically and functionally coupled to the leading-strand replisome.

Unlike lagging-strand synthesis, where p/T junctions are accessible, PCNA loading on the leading strand presents a distinct challenge because the 3′ end is occupied by DNA polymerase ε (Pol ε). How a clamp loader gains access to this DNA substrate has long remained unclear. Ctf18-RFC is specialized for this context. In addition to the canonical AAA+ core, Ctf18 forms an extended “hook” module together with Ctf8 and Dcc1. Earlier structural and biochemical studies showed that this module establishes a stable yet flexible interaction with the catalytic core of Pol ε, tethering Ctf18-RFC to the leading-strand replisome (42) (Fig. 3*A*).

Recent cryo-EM studies now provide near-complete views of the yeast Ctf18-RFC clamp loading cycle (43). While many intermediates resemble those of RFC, several key differences distinguish Ctf18-RFC. First, it lacks a 5′-dsDNA shoulder binding site, indicating that it does not engage gapped DNA in the same manner as RFC. Second, the separation pin is inactive in yeast (Ctf18 Y474), suggesting species-specific modulation of DNA processing (43). Third, the AAA+ module of Ctf18 is more flexible and interacts more weakly with PCNA than Rfc1, consistent with reduced PCNA loading efficiency. These features likely ensure that RFC remains the primary loader during lagging-strand synthesis, whereas Ctf18-RFC can assist leading-strand clamp loading.

A major advance came from the structural analysis of a Ctf18-RFC–PCNA–Pol ε–DNA supercomplex, which reveals how clamp loading is coordinated with the leading-strand polymerase (43) (Fig. 3, *A* and *B*). In this assembly, the hook module positions Ctf18-RFC adjacent to Pol ε, while the P domain and an N-terminal long α-helix, unique for Pol ε but not for other B family Pols, engage the Ctf18 AAA+ module and A′ domain, respectively. These interactions stabilize otherwise mobile regions of the clamp loader, promote an active Ctf18-RFC conformation, and facilitate transfer of DNA from Pol ε to the Ctf18-RFC chamber for PCNA loading (Fig. 3*C*).

These observations support a model in which Ctf18-RFC is recruited to Pol ε *via* the hook module, receives DNA from the polymerase through direct protein–protein interactions, and loads PCNA *in situ*. Following clamp closure, ATP hydrolysis promotes release of Ctf18-RFC, allowing Pol ε to reengage PCNA-encircled DNA for processive leading-strand synthesis. Thus, Ctf18-RFC functions as a polymerase-coupled clamp loader that integrates PCNA loading with replisome architecture.

## Dedicated PCNA unloading by Elg1-RFC

Efficient removal of PCNA from DNA is essential for Okazaki fragment maturation, in which short nascent DNA fragments generated during discontinuous lagging-strand synthesis are processed into a continuous DNA strand, as well as for chromatin organization, and clamp recycling. In yeast, this function is primarily carried out by the Elg1-RFC complex, an alternative clamp loader that serves as the principal PCNA unloader despite comprising only a minor fraction of total RFC-like complexes (49, 50, 68, 69).

**Figure 4:**
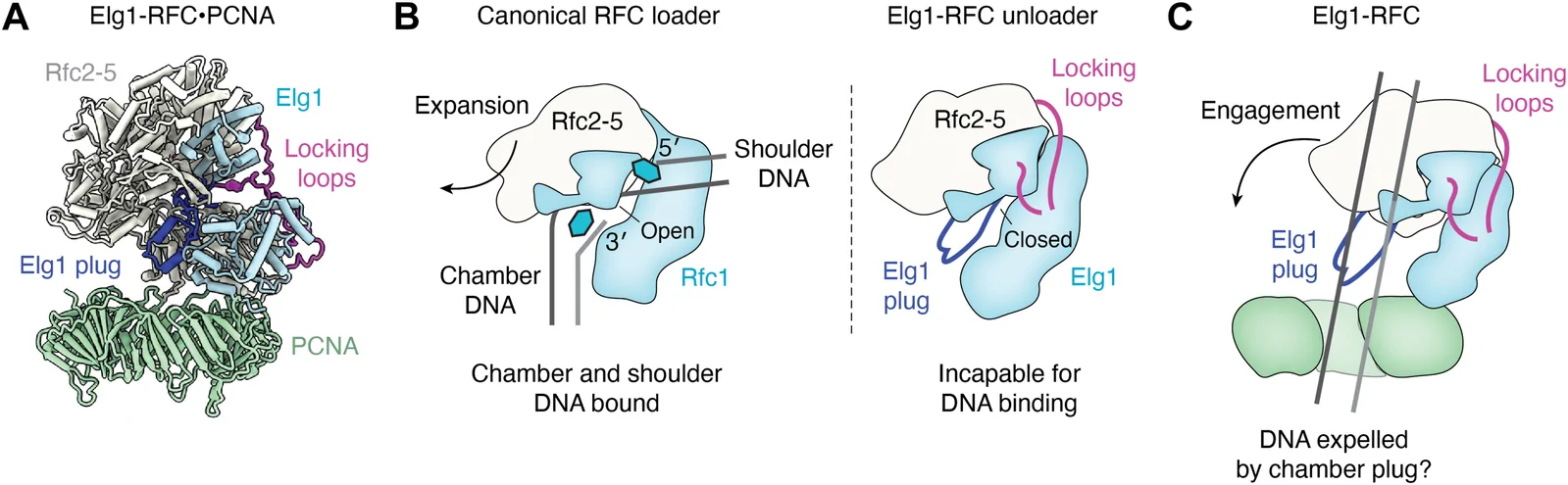
**Structural features that specialize Elg1-RFC for PCNA unloading.***A*, cryo-EM structural studies revealed two striking features in Elg1-RFC–PCNA complex (PDB 8THD); specifically the Elg1 locking loops and the internal plug, which block the shoulder and chamber DNA binding sites, respectively. *B*, in contrast to RFC (*left*), Elg1-RFC (*right*) is incompatible with DNA binding. PCNA molecules associated with RFC and Elg1-RFC are omitted for clarity. In Elg1-RFC, locking loops constrain the A-gate in a closed conformation, whereas the Elg1 plug occupies the central chamber and sterically blocks DNA binding, thereby configuring Elg1-RFC as a dedicated PCNA unloader. *C*, proposed model for PCNA unloading by Elg1-RFC. Progressive interactions with PCNA promote clamp opening, whereas the locking loops maintain a rigid clamp loader conformation and the chamber plug sterically excludes DNA from the complex.

Biochemical and cellular studies established Elg1-RFC as a PCNA unloader, but the structural basis for this activity remained unclear for many years. A recent cryo-EM study has now provided mechanistic insights into PCNA unloading (61). The structures from this study revealed that replacement of Rfc1 with Elg1 remodels the clamp loader into a configuration specialized for unloading rather than loading (Fig. 4*A*).

A defining feature of Elg1-RFC is that it is structurally incompatible with DNA binding. In contrast to RFC, which undergoes a “crab-claw” expansion to open both the clamp loader and PCNA, Elg1-RFC contains two “locking loops” that constrain the A-gate in a closed conformation, preventing the conformational changes required for DNA entry (Fig. 4*B* Left vs Right). In addition, Elg1 harbors a plug element that occupies the central chamber, sterically excluding DNA from binding as speculated (61) (Fig. 4*C*). Together, these features effectively block both chamber and shoulder DNA binding sites, precluding clamp loading activity.

These observations support a model in which Elg1-RFC binds PCNA on duplex DNA after completion of DNA synthesis or repair, induces clamp opening, and expels DNA through steric exclusion, followed by ATP hydrolysis-driven dissociation of the complex. Importantly, this mechanism is fundamentally distinct from, rather than a reversal of, clamp loading; instead, Elg1-RFC operates by excluding DNA while opening and removing the clamp.

Together, these findings establish Elg1-RFC as a specialized PCNA unloader and highlight how remodeling of conserved clamp loader architecture can generate fundamentally different activities.

## Rad24-RFC–mediated 9-1-1 loading restricted to larger DNA gaps

Rad24-RFC is a specialized checkpoint clamp loader that loads the 9-1-1 complex onto recessed 5′ DNA ends to initiate ATR-dependent signaling pathways (44, 45, 46, 47, 48). In contrast to RFC, which loads PCNA at 3′ p/T junctions, Rad24-RFC exhibits strict specificity for 5′-recessed DNA substrates, raising the question of how this selectivity is achieved.

**Figure 5:**
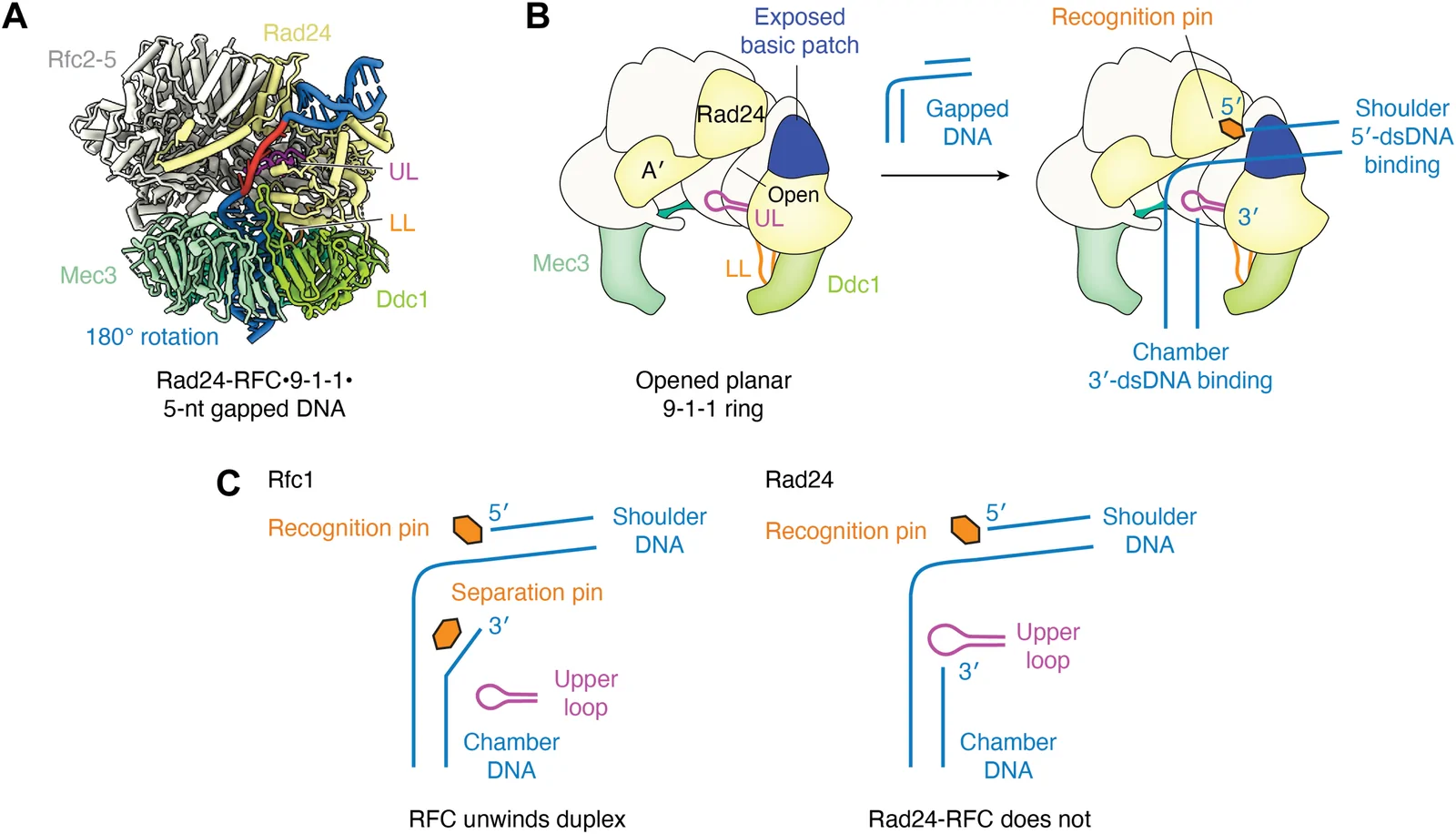
**Rad24-RFC loads the 9-1-1 clamp only onto DNA gaps ≥ 5 nt.***A*, a cryo-EM study revealed that, the 3′-dsDNA in a 5-nt gapped DNA was rotated ∼ 180° to bind the Rad24-RFC–9-1-1 complex (PDB 8FS8), two Rad24 unique features *upper* loop (UL) and *lower* loop (LL) are labeled; see details below. *B*, structural features that define the lower limit of DNA gap size accommodated by Rad24-RFC. Like RFC, Rad24-RFC contains chamber and shoulder DNA binding sites. Unlike RFC, the Rad24 upper loop (UL) and recognition pin restrict the amount of duplex DNA entry into the chamber, limiting efficient loading to gaps ≥ 5 nt. Binding of the Rad24 lower loop (LL) to the 9-1-1 inner chamber is associated with stabilization of an open, planar clamp conformation. *C*, comparison of gapped DNA handling by RFC and Rad24-RFC. RFC can locally unwind the 3′-recessed end through its separation pin to widen small gaps, whereas Rad24-RFC lacks a functional separation pin and instead contains an extended UL that restricts 3′-dsDNA positioning within the chamber, preventing loading onto small gaps.

Cryo-EM studies have provided key insights into the mechanism of 9-1-1 loading (59, 62, 63). These structures show that Rad24-RFC binds 5′-dsDNA at a “shoulder” site formed by an external basic surface on the outside, while also engaging ssDNA within the central chamber rather than dsDNA as observed for RFC (Fig. 5*A*). This finding provides a structural explanation for long-standing biochemical observations of 5′-end specificity, and notably, led to the subsequent discovery of a similar secondary DNA binding site in RFC (34, 35, 36).

Despite this shared architectural feature, Rad24-RFC differs fundamentally from RFC in how it processes DNA. The central chamber of Rad24-RFC is partially occluded by an extended upper loop and the Rfc5 plug, which together prevent duplex DNA from entering the chamber (Fig. 5*B*). In addition, Rad24-RFC lacks a functional separation pin and therefore cannot unwind duplex DNA at recessed 3′-end (Fig. 5*C*). These features impose strict geometric constraints that define substrate selectivity for gapped DNA, a structure that indicates DNA damage.

As a result, Rad24-RFC can only load the 9-1-1 clamp onto DNA substrates with single-stranded gaps. Structural and biochemical data indicate that gaps shorter than 5 nt cannot be efficiently accommodated (60), because the upper loop blocks productive positioning of the 3′-dsDNA within the chamber, and the absence of DNA unwinding prevents widening of the gap. In contrast, larger gaps allow simultaneous engagement of the 5′-dsDNA at the shoulder site and positioning of the 3′-end within the complex.

These observations support a model in which Rad24-RFC first recognizes the 5′-dsDNA at its shoulder site and subsequently positions the 9-1-1 clamp for loading onto appropriately sized gaps. This mechanism contrasts with RFC, which can actively unwind DNA to accommodate small gaps, and thereby establishes a functional division of labor between the two loaders. Rad24-RFC is thus tuned to respond to DNA damage intermediates that generate larger gaps, promoting checkpoint activation, whereas RFC can act on smaller gaps to support DNA repair synthesis (21, 70, 71).

Together, these findings highlight how the structure of Rad24-RFC specifically enforces loading at DNA gaps to ensure appropriate DNA structure-dependent checkpoint signaling.

## Structural diversification of human clamp loaders and unloader

**Figure 6:**
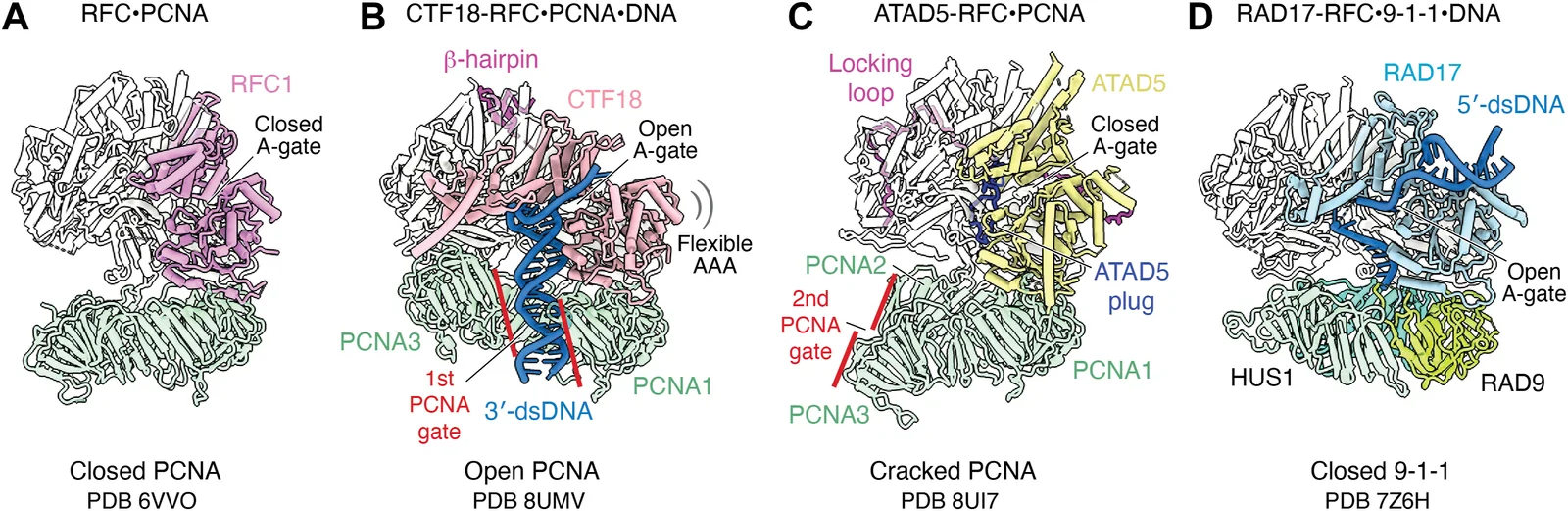
**Cryo-EM structures of human clamp loader and unloader complexes.***A*, structure of the canonical human RFC–PCNA complex in an autoinhibited state, in which RFC1 adopts a closed A-gate conformation and PCNA remains a closed ring. Note there is no DNA bound in this state. *B*, structure of the CTF18-RFC–PCNA–DNA complex bound to p/T DNA. CTF18 adopts an open A-gate conformation and contains a unique N-terminal β-hairpin (magenta, binds to the back of the collar domain) and a flexible AAA+ module, PCNA is opened at the first PCNA gate between PCNA1 and PCNA3 to enable the entry of 3′-dsDNA. *C*, structure of the ATAD5-RFC–PCNA unloader complex. ATAD5 contains a N-terminal long locking loop (*magenta*) that wraps around the unloader, constrains the A-gate in a closed conformation and an internal ATAD5 plug (*blue*) that occupies the central chamber, while PCNA is captured in a cracked state at the unprecedented second PCNA gate between PCNA2 and PCNA3. *D*, structure of the RAD17-RFC–9-1-1 checkpoint clamp loading complex with a 5′-dsDNA bound on the shoulder. RAD17 adopts an open A-gate conformation to accommodate the shoulder 5′-dsDNA, whereas the 9-1-1 heterotrimer remains in a closed ring architecture.

As conserved macromolecular machineries across all domains of life, human RFC and RFC-like complexes operate through principles broadly similar to those of their yeast counterparts, while also exhibiting distinct structural and functional features. Recent cryo-EM studies have identified a few human-specific adaptations that somewhat diversify clamp loading/unloading mechanisms (38, 63, 64, 65, 66) (Fig. 6).

The canonical human RFC–PCNA complex has been captured in an autoinhibited, DNA-free state (38) (Fig. 6*A*). In this structure, RFC1 adopts a closed A-gate conformation, PCNA remains a closed ring, and only the first three subunits (RFC1, RFC3, and RFC4) contact the clamp. This resembles the autoinhibited state observed in yeast RFC–PCNA (30). However, a high-resolution DNA-bound structure of human RFC has not yet been determined, leaving the mechanism of human PCNA loading at p/T junctions or gapped DNA unresolved, *i.e.*, whether it can bind both 3′- and 5′-dsDNA, and also whether the structure-based sequence alignment revealed separation pin (RFC1 W931) can actively melt a DNA 3′-recessed end or not (67).

Besides the similar flexible AAA+ module with yeast Ctf18, human CTF18-RFC presents two different features (43, 65, 66) (Fig. 6*B*). First, CTF18 harbors a unique N-terminal β-hairpin that binds to the back of the collar domain. Second, the separation pin (CTF18 F692) is active whereas the corresponding yeast residue, Ctf18 Y474, is inactive, suggesting species-specific modulation of DNA processing (65).

In human ATAD5-RFC (64), there is only one long N-terminal locking loop that wraps around the complex to constrain the A-gate in a closed conformation, in contrast to the use of two locking loops in yeast Elg1-RFC. Also, the internal plug that occupies the central chamber of human ATAD5-RFC (and sterically excludes DNA) is mainly a loop rather than the two α-helices of the yeast Elg1-RFC internal plug (Fig. 6*C*). Another distinctive feature is that, in the human ATAD5-RFC–PCNA complex, a second PCNA gate between PCNA2 and PCNA3 is observed in addition to the canonical loading gate (between PCNA1 and PCNA2), and this additional gate may facilitate DNA expulsion during unloading.

For the human RAD17-RFC, cryo-EM analysis showed that RAD17 also adopts an open A-gate conformation to accommodate a 5′-dsDNA bound at the external shoulder site (63), while the 9-1-1 clamp remains closed (Fig. 6*D*). This architecture explains the 5′-recessed-end specificity of checkpoint clamp loading and highlights conservation of the shoulder DNA-binding site among eukaryotic checkpoint clamp loaders.

Together, these structures show how alternative large subunits remodel the conserved RFC scaffold to produce distinct activities: canonical RFC is configured as the main PCNA loader for lagging strand DNA synthesis, CTF18-RFC as a specialized PCNA loader for leading strand synthesis with coordination of replisome assembly, while ATAD5-RFC excludes DNA to unload PCNA, and RAD17-RFC recognizes 5′-recessed DNA for checkpoint clamp loading.

## DnaX-mediated DNA bending for β-clamp loading at small gaps

The *E. coli* DnaX complex is the bacterial clamp loader that assembles the β-clamp during DNA replication and repair. Early structural studies established its pentameric architecture (δ:γ_3_:δ′) and revealed how the AAA+ modules form a spiral that engages p/T DNA (29, 31). However, the mechanism by which DnaX loads clamps onto non-canonical substrates, such as nicked or gapped DNA, remained unclear.

Recent cryo-EM studies have captured a near-complete clamp loading cycle of the DnaX complex with β-clamp and DNA (51, 52). These structures reveal a sequence of conformational states in which the clamp transitions from closed to partially open, fully open, and back to closed, delineating the dynamic process of clamp loading. Notably, DnaX opens the β-clamp through a pivot-like motion centered at a single interface, distinct from the more broadly distributed “crab-claw” expansion observed in eukaryotic RFC.

**Figure 7:**
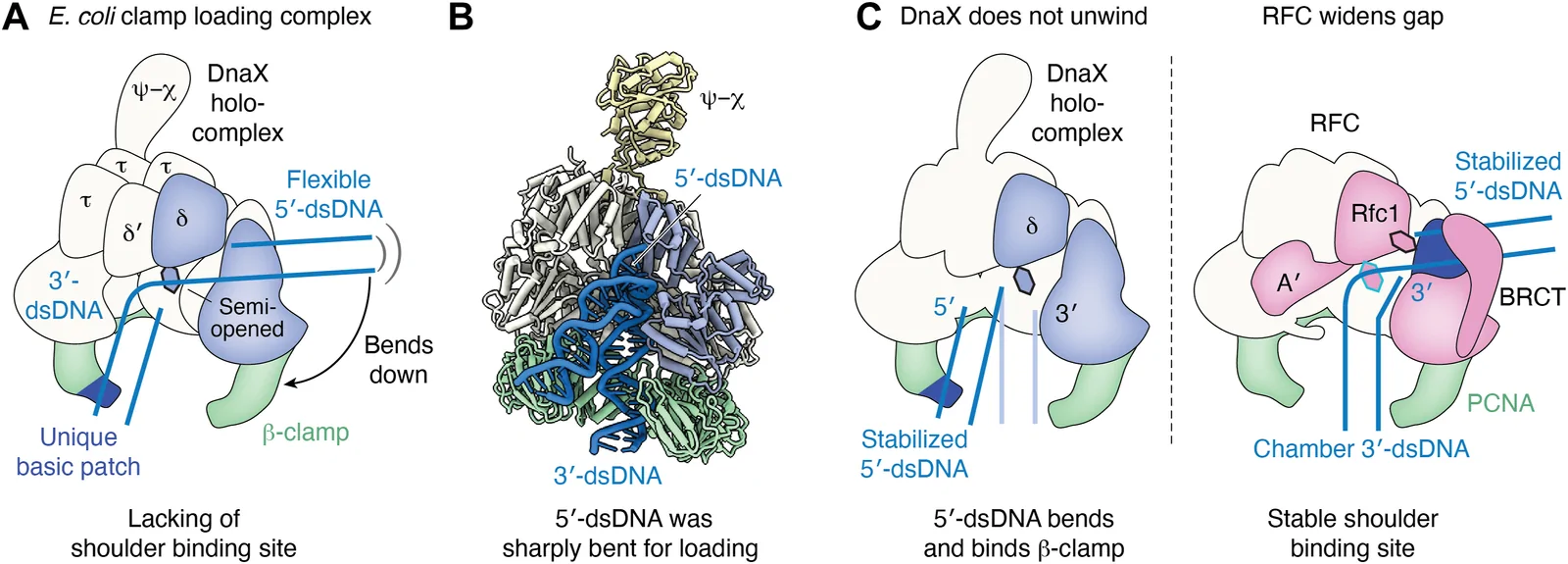
**Distinct strategies used by the *E. coli* DnaX complex and yeast RFC to load clamps onto small DNA gaps (<6 nt).***A*, proposed intermediate in β-clamp loading by the DnaX complex. For clarity, the initial steps in clamp loading—DnaX complex binding to a closed β-clamp (PDB: 8VAN, 8GIY) followed by clamp opening (PDB: 8VAM, 8VAP and 8GIZ)—are omitted. The 3′-dsDNA is first engaged within the clamp loader chamber in a tilted DNA configuration, whereas the 5′-dsDNA remains outside the clamp loader and highly flexible. The 5′-dsDNA will bend down sharply towards the β-clamp basic patch to establish interaction. *B*, cryo-EM structure of DnaX complex and β-clamp bound to a 1-nt gapped DNA. Note the extremely bent DNA which is the state that follows the *panel A* state. Here, the 5′-dsDNA has bent back to bind the β-clamp basic patch, and the 3′ duplex has been “pushed” into the inner chamber (PDB 9OYE). The structure of ψ-χ subcomplex is extracted from another model (PDB 9OYH) to generate a composite model for a complete view. *C*, mechanistic comparison of bacterial and eukaryotic clamp loading at small DNA gaps. In *E. coli* (*left*), the 5′-dsDNA bends sharply back toward the β-clamp without local DNA unwinding. In contrast, yeast RFC (*right*) locally unwinds DNA to widen the gap, enabling simultaneous engagement of 3′- and 5′-dsDNA at the chamber and shoulder binding sites, respectively.

In contrast to RFC and Rad24-RFC, the DnaX complex lacks a 5′-dsDNA shoulder binding site. Structural analysis shows that the corresponding surface region is not positively charged and does not support duplex DNA binding (67). Instead, a basic patch on the β-clamp near the open gate appears to stabilize DNA during the initial stages of loading (Fig. 7*A*). In these intermediates, the DNA adopts a tilted conformation, with the 3′-recessed end engaged within the clamp loader chamber while the upstream 5′-dsDNA remains outside the clamp.

A key mechanistic insight is that DnaX does not unwind DNA to accommodate small gaps. The previously proposed separation pin (δ Y316) is unable to melt duplex DNA, in contrast to its functional counterpart in RFC. Instead, DnaX employs a fundamentally different strategy: the 5′-dsDNA is sharply bent back toward the clamp, allowing it to bind the β-clamp interface as the 3′-dsDNA enters the loader chamber. This bending substitutes for a 5′-DNA binding site and enables clamp loading at very small gaps without requiring strand separation (Fig. 7, *B* and *C*).

These observations support a model in which DnaX first captures the 3′-recessed end in a tilted configuration, stabilized by interactions with the β-clamp, and then bends the 5′-dsDNA to complete clamp loading. This mechanism contrasts sharply with that of RFC, which widens DNA gaps through local unwinding to enable dual-site binding. Thus, bacterial and eukaryotic clamp loaders achieve the same outcome—loading clamps onto constrained DNA substrates—through fundamentally different physical strategies.

## Outstanding questions and future perspectives

Recent cryo-EM studies have substantially advanced our understanding of clamp loading and unloading mechanisms. However, several fundamental questions remain unresolved.

First, what are the molecular events that trigger ATP hydrolysis in clamp loaders? Clamp loading itself requires ATP binding, whereas ATP hydrolysis is thought to promote dissociation of the clamp loader after clamp loading onto DNA. However, the precise triggers for ATP hydrolysis remain unclear and will likely require capturing late-stage intermediates during clamp loading and release.

Second, what is the order of DNA engagement in different clamp loaders? Both RFC and Rad24-RFC contain chamber and shoulder DNA binding sites. Structural studies suggest that Rad24-RFC may first bind 5′-dsDNA at the shoulder site, whereas RFC may initially engage 3′-dsDNA within the chamber (33, 36, 59, 60). However, these models remain hypothetical, particularly for substrates with small gaps where DNA geometry is constrained. A related question applies to the bacterial DnaX complex, for which the sequence of 3′- *versus* 5′-DNA engagement is also unclear.

Beyond these mechanistic questions, it remains unclear how cells determine which clamp loader to deploy at a given DNA intermediate. For example, both RFC and Rad24-RFC are capable of loading their respective clamps onto DNA gaps of intermediate size (∼10 nt), raising the question of how this choice is regulated *in vivo*. Similarly, the biological requirement for clamp unloading remains incompletely understood. Although RFC itself exhibits unloading activity, eukaryotes have evolved a specialized unloader, Elg1-RFC (ATAD5-RFC), whereas bacteria rely on a single clamp loader. Whether checkpoint clamps such as 9-1-1 are actively unloaded, and whether dedicated unloaders exist for these clamps, also remains to be determined.

Finally, how are clamp loading and unloading coordinated in space and time during DNA replication, repair, and checkpoint signaling? Multiple clamp loaders, unloaders, and clamp-binding proteins compete for access to clamps on chromatin, suggesting that these processes must be tightly regulated in cells. Emerging approaches such as cryo-electron tomography (cryo-ET), combined with biochemical and single-molecule studies, will be critical for understanding how these dynamic processes are orchestrated in native chromatin contexts (72, 73, 74, 75).

## Conflict of interest

The authors declare that they have no conflicts of interest with the contents of this article.
